# TWEAK/Fn14 Axis-Targeted Therapeutics: Moving Basic Science Discoveries to the Clinic

**DOI:** 10.3389/fimmu.2013.00473

**Published:** 2013-12-23

**Authors:** Emily Cheng, Cheryl L. Armstrong, Rebeca Galisteo, Jeffrey A. Winkles

**Affiliations:** ^1^Department of Surgery, Center for Vascular and Inflammatory Diseases and Greenebaum Cancer Center, University of Maryland School of Medicine, Baltimore, MD, USA

**Keywords:** TWEAK, Fn14, inflammatory disease, cancer, clinical trial

## Abstract

The TNF superfamily member TWEAK (TNFSF12) is a multifunctional cytokine implicated in physiological tissue regeneration and wound repair. TWEAK is initially synthesized as a membrane-anchored protein, but furin cleavage within the stalk region can generate a secreted TWEAK isoform. Both TWEAK isoforms bind to a small cell surface receptor named Fn14 (TNFRSF12A) and this interaction stimulates various cellular responses, including proliferation and migration. Fn14, like other members of the TNF receptor superfamily, is not a ligand-activated protein kinase. Instead, TWEAK:Fn14 engagement promotes Fn14 association with members of the TNFR associated factor family of adapter proteins, which triggers activation of various signaling pathways, including the classical and alternative NF-κB pathways. Numerous studies have revealed that Fn14 gene expression is significantly elevated in injured tissues and in most solid tumor types. Also, sustained Fn14 signaling has been implicated in the pathogenesis of cerebral ischemia, chronic inflammatory diseases, and cancer. Accordingly, several groups are developing TWEAK- or Fn14-targeted agents for possible therapeutic use in patients. These agents include monoclonal antibodies, fusion proteins, and immunotoxins. In this article, we provide an overview of some of the TWEAK/Fn14 axis-targeted agents currently in pre-clinical animal studies or in human clinical trials and discuss two other potential approaches to target this intriguing signaling node.

## Introduction

The TNF superfamily member TWEAK was first described in 1997 (1), and since that initial publication dozens of research groups throughout the world have studied the biological properties of this multifunctional cytokine. One of the major early milestones in the TWEAK research arena was the cloning of the human TWEAK receptor by Wiley et al. (2), which had 100% predicted sequence identity to a previously reported growth factor-inducible type I transmembrane protein named Fn14 (3, 4). TWEAK is the only TNF superfamily member that can bind Fn14 (5). The TWEAK and Fn14 genes are expressed at low levels in most normal tissues but upregulated in damaged tissue [reviewed in Ref. (6, 7)]. Moreover, the Fn14 gene is highly expressed in both primary tumors (8–14) and tumor metastases (11). TWEAK is synthesized as a type II transmembrane protein but it can be cleaved to generate a soluble cytokine (1, 15, 16). TWEAK:Fn14 engagement promotes TNFR associated factor (TRAF) binding to the Fn14 cytoplasmic tail (17), activation of downstream signaling pathways (e.g., NF-κB, MAPK, PI3K/Akt), and triggering of various cellular responses, including proliferation, survival, migration, differentiation, or death [reviewed in Ref. (6, 7)]. Interestingly, binding of the membrane-bound and soluble TWEAK isoforms to Fn14 can trigger differential downstream outputs; for example, membrane TWEAK is a more potent activator of the classical NF-κB signaling pathway (16).

One important aspect of Fn14 biology that may have clinical implications is the concept of TWEAK-independent Fn14 signaling. Experimental manipulation of Fn14 expression levels in cancer cells cultured *in vitro* can regulate signal transduction and cellular properties; for example, cell migration and invasion (8, 10, 18–20). These findings have led our group to propose that when Fn14 expression in cells reaches a certain threshold level it may signal on its own, even without ligand engagement (6). Recent studies in which we transiently expressed a mutant Fn14 protein that is unable to bind TWEAK support the notion that Fn14 can in fact signal in a ligand-independent manner (21). This signaling mechanism may be particularly important in injured tissues and cancers where Fn14 levels are high but TWEAK levels are low [e.g., in glioblastomas (22) and melanomas (unpublished data)]. We hypothesize that the most likely explanation for TWEAK-independent Fn14 activation is that when Fn14 is expressed at high levels in cells it spontaneously multimerizes, and this will trigger TRAF association, downstream signaling, and cellular responses.

A second critical milestone in the TWEAK-Fn14 research arena was the generation of TWEAK- or Fn14-deficient mice by groups at Genentech (23) and Biogen Idec (24, 25). Studies using these mice, in conjunction with studies testing the effects of TWEAK-neutralizing biologics in mouse models of human tissue injury and disease, have been instrumental in establishing the generally accepted view that TWEAK/Fn14 signaling is important for effective wound repair following acute tissue injury and that chronic Fn14 signaling can promote pathological tissue responses [reviewed in Ref. (6, 7, 26, 27)].

Basic science studies using cells in culture, expression profiling studies using normal and diseased tissue specimens, and *in vivo* studies using wild-type (WT) or genetically engineered mice have all indicated that the TWEAK/Fn14 axis may play an important role in the pathophysiology of several different human diseases [reviewed in Ref. (6, 7, 26–28)]. In general, this axis seems to be primarily involved in disease progression and maintenance, not initiation. Numerous academic and industrial research laboratories have initiated programs to develop biologics or small molecule compounds that activate or inhibit this signaling axis, depending on the disease target [reviewed in Ref. (28)]. Remarkably, the first two TWEAK/Fn14 axis-targeted Phase I clinical trials began recruiting in 2008, only 7 years after the initial report demonstrating that TWEAK and Fn14 were a ligand-receptor pair (2). In this article, we provide an overview of some of the TWEAK- or Fn14-directed therapeutic agents that are presently in pre-clinical development or have entered clinical trials.

## TWEAK/Fn14 Axis-Targeted Therapeutics: Inflammatory and/or Neurodegenerative Diseases

Inflammation is a complex, dynamic process that occurs in tissues following traumatic, infectious, toxic, or autoimmune injury [reviewed in Ref. (29, 30)]. This physiologic response is critical for our ability to heal wounds and fight off pathogens. Inflammation is normally very tightly controlled but when this process is excessive or prolonged it contributes to the pathogenesis of numerous diseases, including atherosclerosis, ischemic stroke, rheumatoid arthritis (RA), and inflammatory bowel diseases [reviewed in Ref. (30–32)]. Persistent TWEAK/Fn14 signaling has been implicated in the pathogenesis of these and other related diseases [reviewed in Ref. (7, 27)] and in this section we summarize some of the TWEAK-targeted therapeutic agents under development for these conditions (Table 1).

**Table 1:** **Examples of TWEAK-targeted therapeutic agents for inflammatory and/or neurodegenerative diseases**.

Agent	Developer	Type of agent	Status	Disease	Reference(s)
Fn14-Fc	EUSOM/UMSOM	Decoy receptor	Pre-clinical	Cerebral ischemia and edema (stroke)	(37, 40, 41)
Fn14-TRAIL (KAHR-101)	KAHR Medical	Signal converter protein	Pre-clinical	EAE (model of multiple sclerosis)	(52, 54)
BIIB023	Biogen Idec	Neutralizing mAb	Phase I trial	Rheumatoid arthritis	ClinicalTrials.gov
			Start date: October 2008		NCT00771329
			Completion date: April 2011		(68)
BIIB023	Biogen Idec	Neutralizing mAb	Phase II trial	Lupus nephritis	ClinicalTrials.gov
			Start date: July 2012 (recruiting)		NCT01499355

*EUSOM, Emory University School of Medicine; UMSOM, University of Maryland School of Medicine; EAE, experimental autoimmune encephalomyelitis; mAb, monoclonal antibody*.

### Fn14-Fc fusion protein

Ischemic stroke ranks third among the most common causes of mortality and is a leading cause of long-term disability in the world (33, 34). The onset of cerebral ischemia triggers a number of pathophysiological events, including oxidative stress, glucose deprivation, disruption of the architecture of the neurovascular unit [which results in an increase in the permeability of the blood brain barrier (BBB) and the development of cerebral edema], and brain inflammation [reviewed in Ref. (32, 34)]. Intravenous administration of the thrombolytic agent tissue plasminogen activator (tPA) within 3–4.5 h of stroke onset is the only FDA-approved treatment for stroke patients (34).

Numerous studies have indicated that TWEAK is a potential novel therapeutic target for treatment of acute cerebral ischemia. Both TWEAK and Fn14 expression have been detected in astrocytes, microglia, endothelial cells, and neurons *in vitro* and *in vivo*. TWEAK expression is more prominent in astrocytes, and Fn14 expression is found predominantly in neurons [reviewed in Ref. (35)]. Early studies using murine models of focal cerebral ischemia showed that both TWEAK and Fn14 mRNA expression in ischemic brain tissue increases within hours after an ischemic insult (36–38). Importantly, there is also an increased level of these mRNAs in human infarct tissue obtained from deceased stroke patients and TWEAK levels are elevated in the serum of stroke patients (39). Functional studies using Fn14-deficient mice have indicated that TWEAK/Fn14 signaling contributes to the cell death and edema that occurs following acute cerebral ischemia (38, 40). Accordingly, Yepes and colleagues tested whether administration of a soluble Fn14-Fc decoy protein shortly after ischemia onset could inhibit TWEAK activity in mouse brain tissue and thereby attenuate cell death and edema. In their initial studies they found that Fn14-Fc injection significantly reduced infarct volume, microglia activation, and apoptotic cell death in the ischemic penumbra (37). Subsequent research found that Fn14-Fc delivery could also improve animal motor activity and reduce the extent of cerebral ischemia-triggered BBB breakdown and edema (40, 41). Thus, TWEAK neutralization may be an effective therapeutic strategy for acute cerebral ischemia.

### Fn14-TRAIL fusion protein

Multiple sclerosis (MS) is an immune-mediated disease of the central nervous system (CNS) that causes progressive neurological deterioration [reviewed in Ref. (42, 43)]. MS is characterized by the presence of focal demyelinated lesions in the white and gray brain matter, the accumulation of inflammatory cells, and BBB disruption with edema. A number of effective anti-inflammatory and immunomodulatory treatments are now available that reduce disease activity for patients in the relapsing-remitting stage of the disease [reviewed in Ref. (43, 44)]. The primary first-line treatments for MS are interferon-β (IFN-β) glatiramer acetate (Copaxone), a synthetic protein whose mechanism of action is not fully understood. Over time, however, autoantibodies can develop in the IFN-β-treated patients, resulting in decreased efficacy. Skin conditions such as erythema were the most common side effects in Copaxone-treated patients [reviewed in Ref. (43)]. If disease stabilization fails to be achieved using these first-line therapies, a number of second-line treatments are available. These second-line treatments leave MS patients at risk for a number of side effects including CNS infection, skin cancer, and cardiac toxicity [reviewed in Ref. (43)]. Therefore, new targeted therapies are needed for MS patients and several studies have indicated that TWEAK deserves consideration as a potential molecular target [reviewed in Ref. (45)].

Experimental autoimmune encephalitis (EAE) is a Th1-mediated autoimmune disease of the CNS that is the most commonly used experimental model for human CNS demyelinating diseases, including MS [reviewed in Ref. (43, 46)]. EAE is induced by inoculating mice with myelin oligodendrocyte glycoprotein (MOG). This leads to a disease course characterized by an acute paralytic phase followed by a moderate remission and finally a chronic phase associated with demyelination. Two independent studies showed that TWEAK/Fn14 pathway inhibition decreased disease severity in the EAE model. In one study, Desplet-Jego et al. found that intraperitoneal administration of an anti-TWEAK-neutralizing monoclonal antibody (mAb) after the priming phase reduced leukocyte CNS infiltration and clinical score (47). In the second study, Mueller et al. showed that intraperitoneal injection of recombinant rat soluble TWEAK and Fn14 extracellular domain resulted in induction of neutralizing antibodies, causing a decrease in CNS inflammatory infiltration and EAE severity in both rat and mouse models (48). It has also been reported that soluble TWEAK overexpression, accomplished either by injection of a TWEAK expression plasmid (48) or the generation of TWEAK transgenic mice (49), increases EAE severity.

Another animal model relevant to MS is cuprizone-induced demyelination. It is an ideal system to study neuroinflammation, demyelination, and remyelination [reviewed in Ref. (46)]. Cuprizone is a copper chelating agent, which causes oligodendrocyte cell death and microglial infiltration into the damaged site in the murine CNS. Whereas in the EAE model demyelination is dependent upon CD4^+^ T cells, demyelination in the cuprizone model is mediated by microglia with little contribution from peripheral macrophages. Iocca et al. showed that modest upregulation of Fn14 and TWEAK occurred during the demyelination phase of cuprizone treatment (50). Furthermore, TWEAK-deficient mice exhibited a delay in demyelination of the corpus callosum after 4 weeks of cuprizone treatment as compared to WT animals.

These studies, in combination with research demonstrating that TWEAK and Fn14 expression is upregulated in human brain specimens from MS patients compared to controls (51) support the contention that TWEAK-neutralizing agents could be effective therapeutics for this disease. In this regard, Razmara et al. reported that transposon-mediated expression of an Fn14-TRAIL fusion protein in mice reduced the incidence and severity of EAE (52). Fn14-TRAIL consists of the Fn14 extracellular domain fused to the soluble form of the TNF superfamily member TRAIL. This “signal converter protein” is designed to both inhibit TWEAK/Fn14 signaling and promote TRAIL/TRAILR signaling, with this second functional attribute included on the basis of earlier work showing that TRAIL attenuates MOG-induced EAE (53). A more recent study by Prinz-Hadad et al. found that purified Fn14-TRAIL protein limited T cell responses and alleviated EAE severity only when administered 10 days after EAE induction as compared to Fn14-TRAIL treatment starting on the day of MOG immunization (54). This suggests that Fn14-TRAIL has a more significant impact on the propagation phase of EAE rather than during the induction phase. Fn14-TRAIL (KAHR-101) is currently under further pre-clinical development by KAHR Medical Ltd., for MS and cancer (see below).

### TWEAK mAb BIIB023

Rheumatoid arthritis is a progressive autoimmune disease characterized by joint inflammation and tissue destruction that leads to disability and a decrease in quality of life. While the cause of RA is unknown, many genetic determinants and environmental factors that contribute to disease susceptibility have been identified [reviewed in Ref. (55, 56)] and the pathogenesis of the disease has been well characterized [reviewed in Ref. (57)]. RA development involves a cellular and humoral immune response to auto-antigens, with patients developing a range of autoantibodies [reviewed in Ref. (58)]. The disease is usually concentrated in the joint regions of the hands, knees, or feet, where the inflamed synovial membrane, caused by activated leukocytes and angiogenesis, contains hyperplastic synoviocytes, pro-inflammatory cytokines, cartilage-degrading enzymes, and the osteoclast-containing pannus that breaks down bone. RA treatment options include corticosteroids, non-steroidal anti-inflammatory drugs (NSAIDs), and disease-modifying anti-rheumatic drugs (DMARDs) such as methotrexate (MTX) and biologics including TNF-α or IL-6 inhibitors [reviewed in Ref. (57, 59)].

Numerous studies have implicated the TWEAK/Fn14 axis as a potential contributor to RA pathophysiology. When TWEAK and Fn14 expression were evaluated by immunohistochemistry of synovial tissue (ST) biopsies, TWEAK expression was higher in patients with RA compared to patients with psoriatic arthritis (PsA) (60) or osteoarthritis (OA) (61, 62). TWEAK levels are higher in serum obtained from RA patients versus control (63) and in both serum and synovial fluid of RA versus OA patients (62, 64). Furthermore, TWEAK expression was detected in newly diagnosed, DMARDs-naive patients as well as TNF-α mAb (Infliximab)-treated patients (60). While Fn14 expression was found elevated in RA patients compared to normal, healthy controls (62) there was no increase relative to PsA patients and no difference between active and inactive RA (60). Co-staining experiments revealed that TWEAK expression was found on fibroblast-like synoviocytes, CD22^+^ B cells, macrophages, plasma cells, multi-nucleated cells, and ST blood vessels (60, 62). *In vitro* studies have shown that TWEAK may contribute to RA pathogenesis through multiple mechanisms such as promoting bone resorption (61, 62) and joint tissue destruction (65), inhibiting bone and cartilage repair mechanisms (65), and promoting joint inflammation (66).

*In vivo* studies using mouse models of RA have shown that the TWEAK/Fn14 signaling pathway is a significant contributor to RA pathogenesis. An initial report showed that TWEAK levels were elevated in the sera of mice with collagen-induced arthritis (CIA) and that the disease severity, assessed by evaluating inflammation in the mouse paws, was decreased when the mice were treated with an anti-TWEAK-neutralizing mAb (65). The TWEAK antibody treatment also resulted in reduced inflammation and reduced cartilage and bone loss according to histological analysis. Furthermore, the TWEAK antibody-treated mice had reduced synovial angiogenesis and a reduction in the serum levels of inflammatory cytokines and chemokines. A second independent study also showed that an anti-TWEAK-neutralizing mAb attenuated the severity of CIA by reducing synovial angiogenesis and inflammatory chemokine levels in the serum and joints of CIA mice (67). Both studies showed that T- and B-cell responses to collagen II were maintained in the CIA experimental animals that were treated with TWEAK antibody, indicating that TWEAK/Fn14 signaling blockade does not have an effect on the adaptive immune response.

Recently, the results of a Phase I clinical trial (ClinicalTrials.gov Identifier NCT00771329) testing the TWEAK-blocking mAb BIIB023 in patients with RA were reported by Wisniacki et al. (68). BIIB023 was well tolerated in RA patients that received a single dose up to 20 mg/kg either alone or as an add-on to TNF-α inhibitor administration, with all patients taking background MTX. The single BIIB023 dose did not increase the incidence of adverse events when added to the TNF inhibitor treatment, and all adverse effects (AEs) were mild to moderate in severity. Additionally, there were no serious infections in this trial, consistent with the idea that blocking TWEAK/Fn14 signaling does not inhibit the adaptive immune response. BIIB023 showed low immunogenicity, favorable pharmacokinetics, and at high doses, was able to suppress serum soluble TWEAK levels for 28 days. BIIB023 is currently under investigation in a Phase II trial in patients with lupus nephritis (LN) (see below).

Systemic lupus erythematosus (SLE) is a chronic autoimmune disease with a relapsing-remitting course and a diverse array of clinical manifestations, including LN, cutaneous lupus, neuropsychiatric lupus, and atherosclerosis, that contribute to a decrease in quality of life and an increase in mortality [reviewed in Ref. (69)]. SLE genetic risk factors and disease pathogenesis have been well studied [reviewed in Ref. (70)]. SLE patients develop antibodies against an array of auto-antigens, including nuclear antigens, which can lead to inflammation and injury in many organs. SLE is primarily treated with glucocorticoids, cyclophosphamide, B cell-targeted therapy (e.g., anti-CD20 mAbs), and immunosuppressive drugs that are associated with a risk of infection, adverse events such as infusion reactions (characterized by fever, chills, rash, and swelling), and organ damage. These serious side effects have lead more recently to the development of targeted, biologic therapies [reviewed in Ref. (59)], but biologic and traditional therapies for SLE are not always effective and there is still a need for the development of novel, targeted therapies.

The TWEAK/Fn14 signaling pathway has been identified as a potential therapeutic target for LN because of its role in promoting inflammation, angiogenesis, cell proliferation, and fibrosis: all biological processes that contribute to LN pathology [reviewed in Ref. (71)]. TWEAK expression has been examined in clinical specimens and while there have been conflicting reports about TWEAK mRNA and protein levels in the peripheral blood mononuclear cells and serum of SLE patients (72–75), there have been consistent reports of elevated TWEAK levels in the kidney and urine of LN patients (73, 76–78). The urinary TWEAK levels in LN patients were found to be increased relative to healthy control donors, SLE patients without LN, RA patients, OA patients, and patients with kidney disease due to diabetes or hypertension. These data suggest that increased urinary TWEAK levels are due to the specific pathology of LN, and not due to a non-specific effect of systemic autoimmunity or decreased kidney function.

Animal studies also support the idea that TWEAK is involved in the local kidney pathology seen in LN. In a chronic graft-versus-host (cGVH) mouse model of SLE, splenocytes are transferred from a donor mouse strain with an MHC class II mutation into a coisogenic recipient mouse. This results in the activation of the alloreactive donor T cells, which in turn costimulate recipient B cells and promote the production of autoantibodies and subsequent kidney damage. When SLE was induced in Fn14-deficient mice, there was a reduction in renal disease as measured by glomerular IgG deposition and urinary protein levels (proteinuria) compared to SLE-induced WT mice (79). There was also a reduction in pro-inflammatory cytokines and chemokines, a decrease in kidney-infiltrating macrophages, and a decrease in renal cellular proliferation. There were no differences in serum IgG levels or serum autoantibody levels between the Fn14-deficient and WT mice, indicating that TWEAK/Fn14 signaling does not play a role in the induction of SLE. When a murine neutralizing anti-TWEAK antibody was used in the same SLE mouse model, a similar reduction in renal disease was observed (79).

In another study using bone marrow chimeric mice, the relative contributions of Fn14 expression in the resident kidney cells and bone marrow in the development of LN were evaluated using the cGVH mouse model of SLE (80). Mice with Fn14^+^ non-bone marrow-derived cells, mice with the Fn14^+^ bone marrow-derived cells, and control non-chimeric cGVH mice displayed the same elevation in serum antinuclear autoantibodies. In contrast, the mice with Fn14^+^ non-bone marrow-derived cells showed a more rapid and stronger nephritis disease course than mice with Fn14^+^ bone marrow-derived cells, as measured by urine albumin levels. These results indicate that Fn14 expression does not impact SLE induction, but that resident kidney cells, and not the bone marrow-derived cells, play a prominent role in LN pathology.

Another model of LN, nephrotoxic nephritis (NTN), was used to evaluate the role of TWEAK and Fn14 in LN pathology (81). NTN is induced by passive transfer of preformed rabbit anti-mouse glomerular antibodies into mice pre-immunized with rabbit IgG, which promotes the deposition of immune complexes and macrophage-mediated injury in the kidney. Fn14-deficient mice with NTN developed reduced renal disease compared to WT mice. Similarly, when NTN was induced in WT mice treated with anti-TWEAK antibody, the mice displayed decreased proteinuria and improved kidney pathology, including a decrease in glomerular proliferation. Additionally, TWEAK antibody-treated mice had decreased mRNA levels of pro-inflammatory mediators, reduced levels of glomerular immune complex deposition, less fibrosis, and less macrophage infiltration of kidneys.

The TWEAK/Fn14 pathway was also shown to promote the neuropsychiatric symptoms of SLE in an MRL lymphoproliferative (lpr) mouse strain that contains a mutation in the Fas gene that results in the spontaneous development of SLE (82). TWEAK and Fn14 mRNA expression were upregulated in the brains of MRL/lpr mice compared to MRL mice and Fn14 protein expression was mostly detected in the endothelial cells. There were no differences found between Fn14-deficient and WT mice in the quantity of a range of autoantibodies. Fn14-deficient mice showed less depression-like behavior in a forced swim test and a saccharin-drink reward test and improved spatial memory in an object placement test, while there was no difference in motor ability detected by a balance beam coordination test. The mRNA expression of pro-inflammatory mediators and RANTES protein expression were decreased in Fn14-deficient brain compared to WT brain. Fn14-deficient mice had decreased CSF albumin relative to WT mice, a measure of BBB integrity. Additionally, anti-dsDNA antibodies, shown to be similar in serum, were increased in the WT brain compared to the Fn14-deficient brain. These data indicate that the TWEAK/Fn14 pathway may promote neuropsychiatric lupus by compromising the BBB.

The anti-TWEAK mAb BIIB023 is currently being evaluated in a Phase II clinical trial (ClinicalTrials.gov Identifier NCT01499355) to test its efficacy against LN, which as mentioned above, is a common manifestation of SLE that can lead to kidney failure. While the primary endpoint of the trial is to test the renal response of LN patients to BIIB023, secondary objectives include assessment of extrarenal SLE disease activity and manifestations [reviewed in Ref. (71)].

## TWEAK/Fn14 Axis-Targeted Therapeutics: Cancer

Cancer is the second leading cause of death in the USA, with ~580,000 deaths predicted to occur in 2013 (83). The vast majority of these deaths are due to metastasis, a complex multi-step process whereby primary tumor cells travel to distant organs and form secondary tumors that can impair critical organ functions [reviewed in Ref. (84, 85)]. Significant progress has recently been made in identifying the molecular genetic abnormalities that drive the growth and spread of some cancer types, and these findings have led to the development of new targeted therapeutics that dramatically improve the survival of those patients with tumors harboring these particular genetic alterations [reviewed in Ref. (86, 87)]. However, the main treatment options for most cancer patients with metastatic disease continue to be chemotherapy and/or radiation therapy. Thus, there is an urgent need to identify new molecular targets for anti-cancer drug development, and in this section we summarize some of the drug development efforts that target the TWEAK and Fn14 proteins (Table 2).

**Table 2:** **Examples of TWEAK- or Fn14-targeted therapeutic agents for cancer**.

Agent	Developer	Type of agent	Target	Status	Comments	Reference
RG7212 (RO5458640)	Hoffmann–La Roche	Neutralizing mAb	TWEAK	Phase I trial	Various cancer types; only patients with Fn14^+^ tumors; no toxicities noted; disease stabilization in some patients	ClinicalTrials.gov NCT01383733 (88, 89)
				Start date: 2011 July	
				Completion date: 2013 March	
Fn14-TRAIL (KAHR-101)	KAHR Medical	Signal converter protein	TWEAK	Pre-clinical	Inhibits tumor growth in xenograft model	(90)
BIIB036 (P4A8)	Biogen Idec	Agonistic mAb	Fn14	Pre-clinical	Inhibits tumor growth in xenograft models	(91, 92)
18D1	UHW/arGEN-X	Agonistic mAb	Fn14	Pre-clinical	Inhibits tumor formation in experimental metastasis assays	(93)
PDL192 (enavatuzumab)	Abbott	Agonistic mAb	Fn14	Phase I trial	Various cancer types; liver and pancreatic enzyme toxicity	ClinicalTrials.gov NCT00738764 (9, 12, 94)
				Start date: 2008 July	
				Completion date: 2011 October	
ITEM4-rGel	MD Anderson Cancer Center/UMSOM	Immunotoxin conjugate	Fn14	Pre-clinical	All three agents inhibit tumor growth in xenograft models	(11, 95, 96)
hSGZ		Immunotoxin fusion protein				(11, 96)
GrB-TWEAK		Ligand-apoptotic factor fusion protein				2013 AACR Meeting Abstract #2185

*UHW, University Hospital of Würzburg; UMSOM, University of Maryland School of Medicine; rGel, recombinant gelonin; GrB, granzyme B; mAb, monoclonal antibody*.

### Strategy #1: Inhibition of TWEAK:Fn14 engagement

#### TWEAK mAb RG7212

Both TWEAK and Fn14 expression has been detected in tumors and TWEAK/Fn14 signaling may promote tumor growth *in vivo* through a variety of mechanisms [reviewed in Ref. (6, 97) and see Ref. (2, 8, 10, 20, 98–100)]; accordingly, agents that inhibit TWEAK binding to Fn14 may have potential therapeutic utility. In a recent report, Yin et al. described the development, functional characterization, and anti-cancer properties of a humanized anti-TWEAK-neutralizing mAb named RG7212 (RO5458640) (88). This antibody blocks TWEAK-stimulated proliferation, NF-κB activation, and cytokine secretion when added to cells cultured *in vitro*. Also, tumor growth inhibition was demonstrated in mouse models in which human cancer cell lines or patient-derived tumorgrafts were xenotransplanted into immunodeficient mice, or alternatively, when syngeneic mouse cancer cell lines were allotransplanted into immunocompetent mice. The greatest tumor growth inhibition was detected in xenograft models of renal cell carcinoma (ACHN, Caki-1), breast carcinoma (MDA-MB-231), and non-small cell lung cancer (Calu-3). TWEAK mAb-treated mice had decreased tumor mRNA levels of various TWEAK-inducible genes and decreased serum levels of mouse (m) MMP9, (m) EGF, (m) bFGF, human (h) CCL2, (h) IL-8, and (h) IL-6. Western blot and immunohistochemistry analyses of tumor tissue showed that TWEAK mAb-treated mice had decreased cell proliferation and increased apoptotic signaling. Depletion of specific immune cells in a syngeneic mouse model showed that CD8^+^ T cells and NK cells, but not CD4^+^ T cells, were required for RG7212-mediated tumor growth inhibition. Taken together, these data demonstrate that TWEAK/Fn14 signaling supports tumor growth by promoting cell proliferation and survival and also by shaping the tumor microenvironment by stimulating the expression of chemokines, cytokines, and inflammatory mediators.

In consideration of the RG7212 pre-clinical data summarized above, a Phase I trial was initiated in 2011 July (ClinicalTrials.gov Identifier NCT01383733). Thirty-eight patients with Fn14-positive tumors were treated with RG7212 (89). No dose-limiting toxicities were observed and most adverse events were considered low-grade. One patient with refractory melanoma showed tumor regression, while another eight patients, including patients with refractory non-small cell lung cancer, renal cell carcinoma, mesothelioma, and metastatic breast cancer, had stable disease for over 3 months. Thus, RG7212 demonstrated tolerability and some efficacy in patients with advanced solid tumors.

#### Fn14-TRAIL fusion protein

The Fn14-TRAIL protein described above in the context of MS has recently been shown to induce hepatocellular carcinoma (HCC) cell apoptosis *in vitro* in a dose-dependent manner (90). In contrast, the fusion protein did not have a significant impact on cell viability in non-malignant hepatocyte cell lines. Interestingly, HCC cells treated with Fn14-TRAIL underwent higher rates of apoptosis than when treated with soluble TRAIL or soluble Fn14 extracellular domain either alone or in combination. Furthermore, subcutaneous administration of Fn14-TRAIL significantly inhibited HCC xenograft growth *in vivo* (90). It is unclear at his time whether TWEAK neutralization is contributing to the anti-tumor effects noted with this agent.

#### Comments on strategy #1

The main concern with this strategy is that TWEAK is expressed at low levels in many Fn14-overexpressing tumors and in this situation it is likely that TWEAK-independent Fn14 signaling occurs, which will not be inhibited by these agents.

### Strategy #2: Activation of the Fn14 signaling pathway

#### Fn14 mAbs BIIB036, 18D1, and PDL192

Fn14 gene expression is elevated in over a dozen different solid tumor types compared with matched adjacent normal tissue or normal tissues from non-diseased donors (8–14); consequently, Fn14 is also a potential target for cancer therapy. TWEAK is a pro-apoptotic factor for some human cancer cell lines [reviewed in Ref. (6, 101)] and several studies have revealed that TWEAK-stimulated cell death occurs via an indirect mechanism; in particular, via TWEAK-triggered upregulation of other cytokines [e.g., TNF-α (102–105) and IFN-β (106)]. The ability of TWEAK to promote tumor cell death has provided the rationale for several groups to explore the potential of agonistic Fn14 mAbs as anti-cancer therapeutics. Scientists at Biogen Idec have developed a humanized anti-Fn14 antibody named BIIB036 (P4A8) that binds with high affinity to Fn14 in BiaCore and FACS assays (91). BIIB036 treatment of Fn14^+^ human cancer cell lines activated the NF-κB signaling pathway, stimulated IL-8 production, and in some cases induced cell death, indicating that this mAb exhibits agonistic activity. This mAb can also inhibit the binding of both membrane-bound and soluble TWEAK to Fn14 (91, 107). Intraperitoneal injection of BIIB036 inhibited WiDr colon carcinoma, NCI-N87 gastric carcinoma, MDA-MB-231 breast cancer, and patient-derived colorectal tumor cell growth in xenograft assays (91, 92). Subsequent studies indicated that the optimal dosing for BIIB036 in tumor-bearing mice was twice weekly and tumor growth inhibition persisted up to 50 days after the termination of dosing (92). Finally, BIIB036 exhibited antibody-dependent cell-mediated cytotoxicity (ADCC) *in vitro* and maximal BIIB036 anti-tumor activity *in vivo* required full Fc effector function, indicating that ADCC was one of the mechanisms mediating the observed inhibitory effects (91). BIIB036, as well as the 18D1 and PDL192 mAbs described below, exhibits the strongest agonistic activity upon oligomerization with protein G or binding to Fcγ receptors (93, 107).

Researchers at the University Hospital of Würzburg and arGEN-X recently reported the generation and characterization of three llama-derived anti-Fn14 mAbs (93). Two of these mAbs, 18D1 and 4G5, blocked TWEAK:Fn14 binding and all exhibited agonistic activity (including cell death induction in the presence of cycloheximide). The 18D1 antibody inhibited metastatic colony formation in both RENCA cell and HCT116 cell experimental metastasis assays, and the anti-metastatic effect noted in the RENCA cell experiments was predominantly mediated via ADCC.

Facet Biotech, which was acquired by Abbott Laboratories in 2010, has also developed an agonistic Fn14 antibody for potential therapeutic use (9). The murine form of this antibody, 19.2.1, was shown to inhibit the growth of multiple cancer cell lines in a dose-dependent manner, including HSC3 oral squamous carcinoma cells, A253 salivary gland cells, A375 melanoma cells, and MiaPaCa2 pancreatic cells. Its humanized counterpart, PDL192 (Enavatuzumab), also exhibited growth inhibitory activity *in vitro* (9). Studies using HER2^+^ SKBR3 breast cancer cells revealed that PDL192 had a synergistic effect on growth inhibition when combined with trastuzumab (Herceptin), the standard treatment for HER2^+^ breast cancer patients (12). PDL192 also exhibited anti-tumor activity in multiple xenograft models of breast cancer, including the MCF-7, HCC70, and MDA-MB-231 models (12). However, the antibody had little to no effect on the *in vivo* growth of other Fn14^+^ cell lines, including Calu6 lung cancer cells and HCT116 colorectal cancer cells (9). As found for BIIB036, the observed growth inhibitory effects of PDL192 on xenograft tumor growth in mice are mediated, at least in part, by ADCC (9). In contrast to BIIB036 and 18D1, PDL192 does not recognize murine Fn14 and does not interfere with TWEAK:Fn14 binding (93, 107). Abbott Laboratories initiated a Phase I clinical trial in 2008 July to test the effects of PDL192 administration (six dosage levels) in 30 patients with advanced solid tumors (ClinicalTrials.gov Identifier NCT00738764). The trial was completed in 2011 October and the results were disclosed in Abstract form at an international meeting (94). No patients showed partial or complete tumor regression but two patients had stable disease for 2–4 months duration. However, patients treated with PDL192 at doses demonstrating anti-tumor efficacy in mice had elevated liver and pancreatic enzyme levels, indicating significant drug toxicity in these organs.

#### Comments on strategy #2

Monoclonal antibodies that target specific cell surface receptors on tumor cells may attenuate tumor growth *in vivo* via multiple mechanisms, including receptor activation, blockade of ligand:receptor engagement, and stimulation of immune cell Fc receptor-mediated pathways such as ADCC. All three of the Fn14-specific mAbs described above have complex biological properties when characterized *in vitro* (93, 107) and thus the precise mechanisms responsible for their apparent anti-tumor growth (PDL192, BIIB036) and anti-metastatic (18D1) activity is not clear. The main concern with the use of Fn14 mAbs that may exhibit agonistic activity within the tumor milieu is that Fn14 activation in most “normal” and cancer cell lines does not cause cell death. Indeed, Yin et al. found that TWEAK treatment had no effect on the viability of 299 tumor cell lines as assessed by CellTiter-Glo or MTT assays (88). In general, most studies support the view that TWEAK/Fn14 signaling in the tumor microenvironment will most likely stimulate pro-tumorigenic/metastatic cellular responses, including tumor cell growth (88, 108), invasion (8, 10, 18–20), and resistance to chemotherapeutic agents (109). There is also evidence that TWEAK can act on Fn14^+^ endothelial cells and promote angiogenesis (2, 100, 110); thus, if TWEAK is expressed by tumor cells or tumor-associated stromal/immune cells *in vivo* it could promote tumor vascularization. Agonistic Fn14 mAbs could have a similar biological effect.

### Strategy #3: Direct killing of Fn14-positive cancer cells

#### Fn14 mAb-based immunotoxins

Targeted toxins are a class of cancer therapeutics that internalize into malignant cells to deliver a cytotoxic payload. These agents consist of a targeting polypeptide (e.g., an antibody, an antibody fragment, or a receptor ligand) that is either chemically conjugated or covalently linked to a toxin, which is usually of bacterial or plant origin [reviewed in Ref. (111, 112)]. Targeted toxins that utilize a mAb or the variable fragment (Fv) of an antibody as the targeting moiety are generally referred to as immunotoxins. Typically, targeted toxins bind specific cell surface receptors, become internalized via receptor-mediated endocytosis, and then the toxin moiety is eventually delivered to the cytosol, its site of action. Immunotoxins targeting a variety of cell surface proteins are currently being evaluated in human clinical trials [reviewed in Ref. (112)].

Two types of Fn14-targeted immunotoxins have been developed and tested in pre-clinical cancer studies by Rosenblum and colleagues (11, 95, 96). Both agents are based on the anti-Fn14 antibody ITEM4 and the plant toxin gelonin. ITEM4 was raised against the human Fn14 extracellular domain (113). It binds both human and murine Fn14 and has weak agonistic activity (11, 113, 114). Gelonin is a ~29-kDa *N*-glycosidase found in the seeds of *Gelonium multiflorum* plants that cleaves 28S rRNA and thereby inhibits protein synthesis (115). The first immunotoxin, ITEM4-rGel, was composed of the ITEM4 mAb chemically conjugated to a recombinant form of gelonin (rGel) purified from *E. coli* cells. This agent was highly cytotoxic when added to Fn14^+^ cancer cell lines *in vitro* and inhibited human T-24, MDA-MB-435, and MDA-MB-231 cancer cell growth in xenograft assays after intravenous administration (11, 95, 96). The second immunotoxin was a humanized, bivalent single-chain protein named hSGZ. The N-terminal region of this protein consists of a single-chain Fv (scFv) fragment of ITEM4 in which the *V*_H_ and *V*_L_ sequences from the hypervariable region were linked by a flexible peptide. These two domains were “humanized” by site-specific mutagenesis to alter certain amino acid residues within the framework domain without impacting affinity or specificity. The C-terminal region consists of rGel followed by a short dimerization domain to produce a bivalent immunotoxin. Recombinant hSGZ protein purified from *E. coli* cells is also highly cytotoxic to Fn14^+^ cells *in vitro* and therapeutic efficacy studies showed significant growth inhibition in both MDA-MB-435 melanoma and MDA-MB-231 breast cancer xenograft models (11, 96). No overt toxicity was noted in mice treated with either the ITEM4-rGel or hSGZ immunotoxins, although as mentioned above, ITEM4 can bind murine Fn14 (114). Thus, these two immunotoxins appear to be effective anti-cancer agents in pre-clinical studies.

#### Granzyme B-TWEAK fusion protein

A completely human fusion protein designed to kill Fn14^+^ cancer cells was recently described by Zhou et al. (96) at the 2013 AACR Annual Meeting (Abstract #2185). This construct, named granzyme B (GrB)-TWEAK, uses the human TWEAK receptor-binding domain as the targeting moiety and GrB as the cell-killing agent. GrB is a serine protease stored in the secretory granules of cytotoxic T lymphocytes and NK cells [reviewed in Ref. (116, 117)]. It normally enters target cells (e.g., non-self or virally infected) via the granule exocytosis pathway and promotes apoptotic cell death [reviewed in Ref. (116, 117)]. GrB-TWEAK is expressed in transfected HEK293 cells as a catalytically inactive protein, purified from conditioned media using nickel-NTA affinity chromatography, and then activated by *in vitro* incubation with enterokinase. Initial studies have revealed that GrB-TWEAK is rapidly internalized by Fn14^+^ cancer cells and is highly cytotoxic in the low nanomolar range. Also, GrB-TWEAK administration (iv) inhibited HT-29 colon carcinoma cell growth in immunodeficient mice (Abstract #2185 Poster).

#### Comments on strategy #3

Importantly, the therapeutic efficacy of these agents does not depend on promoting or inhibiting intracellular signaling pathways or on immune cell function (i.e., ADCC). Also, since these agents directly kill cancer cells, vulnerable cells cannot activate compensatory signaling cascades that promote drug resistance. Nevertheless, there are a number of obstacles that limit the clinical application of this class of therapeutic agents; for example, bacterial or plant toxins can cause vascular leak syndrome and elicit immune responses in patients [reviewed in Ref. (112, 118)]. However, with respect to rGel immunogenicity, in a recent Phase I trial in which an anti-CD33 mAb-rGel immunoconjugate was administered to 28 patients with advanced myeloid malignancies, the immunogenicity of the rGel moiety appeared to be low, as only 2 patients developed detectable levels of anti-rGel antibodies during the course of treatment (119).

## TWEAK/Fn14 Axis-Targeted Therapeutics: Additional Approaches

Protein-based therapeutics like those listed in Tables 1 and 2 can only act on cell surface or secreted molecules, are costly to produce, and have other inherent disadvantages; for example, they generally have to be administered intravenously and they have poor tissue penetration [reviewed in Ref. (120)]. Here, we describe two additional classes of therapeutic agents that may be suitable for inhibition of chronic TWEAK/Fn14 signaling in diseased tissue.

### RNAi-based therapeutics to down-regulate TWEAK or Fn14 gene expression

RNA interference (RNAi) is a potent mRNA sequence-specific post-transcriptional gene silencing mechanism that is currently being evaluated for use as a therapeutic strategy for a wide range of human disorders [reviewed in Ref. (121, 122)]. The goal is to down-regulate the expression of cellular genes involved in disease by administration of either synthetic small interfering RNAs (siRNAs) or gene constructs encoding short hairpin RNAs (shRNAs). Numerous siRNA/shRNA therapeutics have already advanced into clinical trials [reviewed in Ref. (123)], but there are still significant challenges to overcome for safe and effective use of these agents. Some of these challenges include ineffective delivery into target cells, the potential for off-target effects, and immune system-mediated toxicities [reviewed in Ref. (121, 122, 124)]. Although there have been no reports to date describing TWEAK or Fn14 depletion *in vivo* after localized or systemic delivery of either siRNA duplexes or shRNA constructs, this is an approach that should be considered in the near future as progress continues to be made on the development of RNAi-based drugs for treatment of human diseases.

### Small molecule therapeutics to inhibit TWEAK trimerization, TWEAK:Fn14 binding, Fn14 multimerization, or Fn14:TRAF interaction

The classic model of TNF:TNFR superfamily signaling is that the binding of homotrimeric ligands to receptor monomers promotes receptor trimerization (and most likely multimerization), which in turn leads to the recruitment of intracellular adaptor proteins to the cytoplasmic domains of the receptors. Thus, effective signal transduction depends on a series of highly specific protein-protein interactions. Although targeted disruption of protein-protein interfaces by small organic molecules is generally regarded as a challenging goal [reviewed in Ref. (125)], there have been some successes with this approach in the TNF/TNFR superfamily field. For example, compounds have been identified that bind to trimeric TNF-α (126) or CD40L (127), thereby causing conformational perturbations that inhibit receptor binding and functional activity. Small molecule inhibitors of the TNF-α:TNF-R1 interaction have also been reported (128).

TWEAK/Fn14 pathway-specific small molecule inhibitors could potentially be identified using several experimental strategies, including: (i) the use of cell- or recombinant protein-based high throughput screening assays for testing of synthetic chemical or natural product libraries, and (ii) computational methods such as virtual screening using X-ray- or NMR-derived protein structures and chemical compound databases (*in silico* drug discovery). In regard to the first strategy, a high throughput screen measuring the effect of ~60,000 compounds on soluble TWEAK binding to the Fn14 extracellular domain was conducted by Benicchi and colleagues (129). Fifteen primary hit compounds were identified and subsequent counterassays revealed that two were potent but partial inhibitors and two were weak inhibitors (overall hit rate of 0.007%). However, the inhibitory activity of these molecules was not confirmed using other types of assays, and experiments were not conducted to determine the actual protein target (TWEAK or Fn14). In regard to the second strategy, structures of the human Fn14 extracellular domain (130, 131) and of the soluble TWEAK trimer (in complex with the Fab fragment of a neutralizing mAb) (132) have been determined via NMR and x-ray crystallography, respectively. This information, in conjunction with TWEAK (131, 133) and Fn14 (131, 134) mutagenesis data and the available structures of several closely related TNF:TNFR superfamily member complexes, has been used by three groups to derive molecular models of the TWEAK:Fn14 complex (131–133). Dhruv et al. (133) successfully utilized their validated model for the *in silico* identification of small molecule inhibitors of TWEAK:Fn14 binding. Briefly, 60 compounds scoring positive in the virtual screen were obtained and rescreened for inhibitory activity using an ELISA assay. Subsequent studies indicated that one of the compounds, L524-0366, was a potent inhibitor of TWEAK-stimulated NF-κB pathway activation and glioma cell migration *in vitro*. It is anticipated that more small molecule drugs disrupting either TWEAK:Fn14 binding or other critical protein-protein interactions associated with this signaling node will be discovered in the next few years.

## Summary

**Figure 1:**
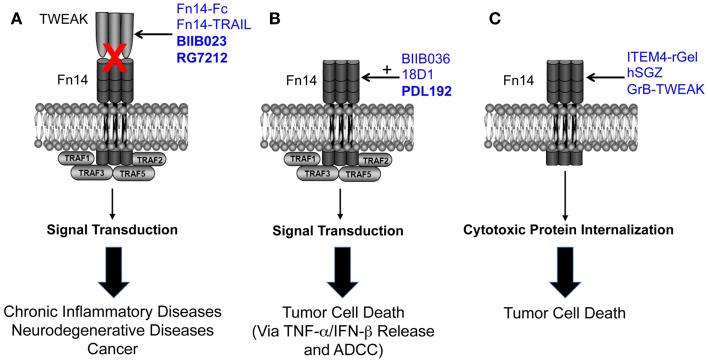
**Summary of the three TWEAK/Fn14 axis-targeted therapeutic strategies discussed in this review**. **(A)** Four TWEAK-neutralizing agents have been developed to prevent TWEAK:Fn14 engagement and thereby inhibit disease progression. **(B)** Three Fn14-directed agonistic mAbs have been developed to activate Fn14 signaling in tumor cells and promote cell death. Studies have shown that TWEAK-induced cell death *in vitro* is an indirect effect mediated by other cytokines, so the anti-tumor efficacy of these agents may occur via this mechanism as well as by ADCC. The agonistic activity of these mAbs is potentiated by oligomerization with protein G or binding to Fcγ receptors. BIIB036 and 18D1, but not PDL192, can also inhibit TWEAK:Fn14 binding. **(C)** Three Fn14-directed agents have been developed that use Fn14 as a portal to deliver toxins or pro-apoptotic proteins into tumor cells. All of the agents shown in this figure have therapeutic efficacy in animal models, and the three agents in bold type have been or are currently under investigation in human clinical trials. Drawing adapted from Figure 3 in Ref. (135).

The TWEAK/Fn14 axis was first described ~12 years ago, and since that time studies from around the globe have revealed that Fn14-triggered signal transduction and downstream cellular responses could potentially play a role in the pathophysiology of many major human diseases, including cardiovascular disease, stroke, and cancer. These studies have led to drug development efforts and the first TWEAK- or Fn14-targeted therapeutic agents (Figure 1). Three of these agents have already entered clinical trials, and this is remarkable progress. However, only the collaborative efforts of basic, translational and clinical researchers will continue to move the TWEAK/Fn14 axis field forward in the coming years, and it is anticipated that this research will ultimately lead to safe and effective therapeutics for clinical use.

## Conflict of Interest Statement

The authors declare that the research was conducted in the absence of any commercial or financial relationships that could be construed as a potential conflict of interest.
